# Peer Review of “Human Brucellosis in Iraq: Spatiotemporal Data Analysis From 2007-2018”

**DOI:** 10.2196/60433

**Published:** 2024-07-03

**Authors:** Anonymous

**Keywords:** human brucellosis, livestock, clustering, spatial, temporal, Iraq

*This is the peer-review report for “Human Brucellosis in Iraq: Spatiotemporal Data Analysis From 2007-2018.”*

## Round 1 Review

### General Comments

This paper [1] presents a spatiotemporal distribution analysis of the outbreak of the brucellosis in Iraq from 2007 to 2018, providing explanations for potential underlying causes. The methods employed include descriptive analysis and Getis-Ord *G*_i_*. The paper exhibits a well-structured format, clear language, rich content, and appropriate methodology.

### Specific Comments

#### Major Comments

1. The *Abstract* and the main text exhibit inconsistency in describing the methods employed. The *Results* section of the main text only includes the results of the descriptive analysis and Getis-Ord *G*_i_*, with no mention of the Moran *I* method as indicated in the *Abstract*.

2. The methods used in the paper should be briefly explained in the *Method*s section to clarify their principles.

3. In the *Results* section, the authors state that there is an increasing trend in female cases from 2016 onward. This conclusion cannot be drawn; female cases increased from 2016 to 2017 and then decreased by 2018, falling below the 2016 quantity.

4. Include spatial distribution maps of the incidence rates for 1-2 years during the study period.

#### Minor Comments

5. Add numerical labels to the bars in Figure 1 for a more intuitive understanding.

6. Figure 4 lacks coordinate axes, and there is an incomplete gray box on the horizontal axis, affecting aesthetics.

7. Please provide the formula for calculating the case frequency.

## Round 2 Review

### Specific Comments

#### Major Comments

1. Maybe I did not express it clearly, but for the local Getis-Ord *G*_i_* method, which is one of the main methods applied in this paper, the authors should give the formula for its calculation and add the source.

2. This is not a comment that has to be revised. Generally, the significance and spatial location of clusters in the local Getis-Ord *G*_i_* results are shown on the same map; for example, hot spots with different levels of significance are represented by 3 progressively deeper red colors, and cold spots with different levels of significance are represented by 3 progressively deeper blue colors. Also, Figure 5 contains too many maps, and it is more concise to show the results for 1 year in 1 map.

3. The elements that are really necessary inside a map, including but not limited to a scale, a compass, and preferably the addition of national boundaries, are missing.

#### Other Comments

The authors have finished revising, and I do not have any questions.
